# The Benefits of Robotic Surgery: Are They Technical or Molecular?

**DOI:** 10.1007/s11605-020-04901-2

**Published:** 2021-01-06

**Authors:** John C. Alverdy

**Affiliations:** Department of Surgery, University of Chicago, 5841 S Maryland MC 6090, Chicago, IL 60647 USA

## Introduction

Training to be a commercial pilot for a major airline today requires inordinate hours of practice in a high-tech simulator and multiple hours of actual air flight in a high-tech commercial air plane. The formal and rigorous certification process and training, coupled with the many technological advances in airplane design and equipment, have made commercial air flight safer today than ever. Yet to specifically determine the influence of pilot technical competence on air flight complications, which in this example would be passenger injury or death, is problematic. The reason for this is uncoupling the pilot’s technical competence from the airplane’s advanced enabling technology, built in environmental redundancies (i.e., checklists, a co-pilot, communication to the tower, GPS, etc.) and is challenging, if not impossible. Short of an individual pilot safely landing a commercial jet with 100 passengers or more in the Hudson river with no casualties while disengaged from all enabling technology and communication, it is becoming increasingly challenging, if not impossible to single out the individual as the independent operational variable of a complex task that involves advanced instrumentation and team cooperation. Analogously, emerging technology in robotic surgery may be similarly challenged as the “captain of the ship” notion as being responsible for all outcomes fades as dependence on instrumentation and a functioning team becomes more integral to the outcome. From the patient’s viewpoint, in terms of safety and efficacy, one might consider this shared responsibility an advance. In this piece, we posit that when examining the processes, executions, and advantages of robotic surgery, one might consider that something else beyond the instrument itself is at play. Here we attempt to outline this notion by positing that beyond technique, there may be multiple positive elements that contribute to the improved outcomes being observed with robotic surgery inclusive of more stringent patient selection, mandated procedure rehearsal, a greater attention to operating bloodlessly, a smoother anastomotic construction beyond the technical aspects of the connection itself, and a higher demand on oneself to operate at peak performance. While invariably there will be multiple attempts to uncouple each element to better understand how the whole process of robotic surgery is an advance at multiple levels, here we assert that such an exercise is both unnecessary and impossible.

## The Flaw of Dismissing the “Carryover Effect” When Assessing Outcome in the Modern Era of Surgery

Due to improvements in patient selection, technique, instrumentation, and anesthesia, there is little doubt that surgery is safer today compared to previous times. In all cases, early capture of disease though surveillance and imaging, the complex planning of surgery, advanced instrumentation leading to less tissue trauma, and attention to early discharge via enhanced recovery programs, in the aggregate, have resulted in improved outcomes. Relative to surgery decades previously, for all cases, open or otherwise, patients are doing better, eating sooner, and being discharged from the hospital at unprecedented earlier times in the postoperative period. Having now practiced for 30 years, it strikes this open-trained, self-taught laparoscopist who now performs most cases robotically, that we are approaching a new era in surgery that not only benefits from this “carryover effect”, but also benefits from emerging technology in both instrumentation and processes of care. Similar to commercial air flight, attempting to uncouple and independently weigh each of the contributing variables involved in this response is not only unnecessary but often may be impossible as one tries to separate the sum of the parts from the whole.

## The Fallacy of Division or Simpson’s Paradox^1^ When Evaluating the Benefits of Robotic Surgery

It is hard for us to imagine that someday, we will be able to analyze wine or cheese for their individual components and somehow they can be made to taste equally delicious were the components reassembled in a chemistry laboratory. Given the complexity involved in producing a high-quality wine or delicious cheese, the concept that the whole is greater than the sum of its parts can be similarly applied to the complexity of robotic surgery. Here I posit that there is some type of “chemistry” at play with robotic surgery that cannot be divided up into its individual parts and simply reassembled (i.e., fallacy of division) to produce the desired effect. Is it the manner of tissue handling, the contemplative and rehearsed aspect of robotic surgery, the “Feng shui” of the operating room, the heightened attention to the presence and dependence on technology, greater direct nursing participation, robot company representatives, the more detailed and serious nature of obtaining the consent, the ability to educate trainees in a much less tense environment, etc. that make the outcome of robotic surgery more favorable than other techniques? As might be imagined, separating each component from the whole and comparing them to other approaches without uncoupling the other embedded elements and carryover effects of accumulating knowledge and practice in surgery presents a major challenge. To this aging surgeon, the best of surgery is here and robotic surgery is ineluctably baked into its future. To me, robotic surgery is better than all other forms, yet proving it better may neither be a hypothesis worth testing nor one that can be tested. Given that enabling technology and more defined structure in training and education have been an ever-present part of our history and are the very reasons that today surgery is safer and more effective than ever, issues of cost-effectiveness may be as irrelevant as justifying driving a car with an automatic transmission, lane indicators, GPS, etc.

## Complication Rates Are Reported to Be Lower with Robotic Surgery: Is This Benefit Technical or Molecular?

Today, serious bleeding requiring blood transfusion after elective surgery is rare. In fact today, transfusion rates following elective surgery are lower than in any time in history^2^. Furthermore, with robotic surgery, bleeding complications are reported to be even less when compared to all other forms of surgery.^3^ While it is natural to assume that this must be a function of better visualization, more precise technique, advanced vessel sealing, etc., molecular factors may also be at work including subtle changes in the coagulation system as a result of a more rigorous planning, timed cadence, tissue handling, more stringent acceptance of any bleeding, etc. Similarly, it is reported that anastomotic leak rates are lower with robotic surgery compared to all other forms of surgery.^4^ Again, despite the prevailing bias and unsubstantiated claim that most anastomotic leaks are due to an error in surgical technique, emerging evidence of a more molecular basis to anastomotic leaks lends plausibility that lower leak rates with robotic surgery may extend beyond its potential technical advantages, be they real or perceived.^5^ For example, the recent discovery that elements with the coagulation system (i.e., plasminogen/plasmin) play a role in anastomotic leak pathogenesis may offer a molecular explanation for lower bleeding and leak rates with robotic surgery.^6, 7^

**Fig. 1:**
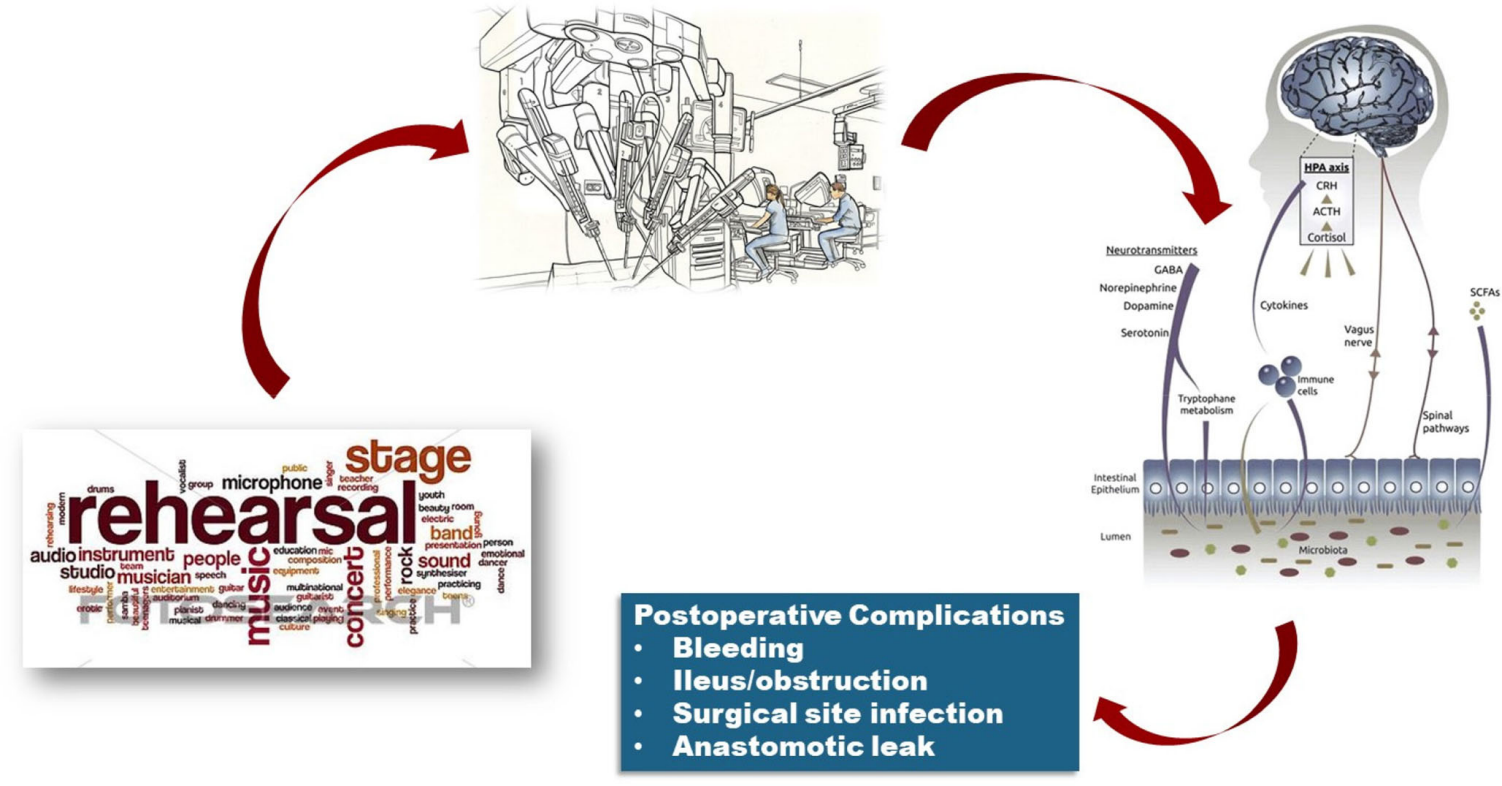
Orchestration, rehearsal, and performance aspects of robotic surgery and its effect on the neuroendocrine immune system and microbiome. As with any high-quality orchestrated, rehearsed, and choreographed performance, robotic surgery may demand a level of team coordination and environmental excellence that invokes yet-to-be identified “molecular” effects on patient recovery and outcome

The above discussion begs the question: is there some molecular aspect of robotic surgery that cannot be measured that drives its benefits beyond technique? Rehearsals prior to a given procedure to the point where it is deemed “performance-ready” may play a role. For example, it may be worth recalling that surgeons operate in a “theater” in which a certain level of self-congratulation as well as self-criticism is expected. No doubt in this scenario, experience matters and it may be for this reason that high-volume surgeons working in high-volume centers who perform a limited but focused repertoire of procedures seems to be associated with superior outcomes.^8, 9^ Yet somehow, robotic surgery and its process of training and expectation of proper attention to the steps, timing, instruments, and cadence (i.e., its orchestration) seems to demand a master performance for each case. Does the structure and process of certification that is now part of the governance of robotic surgery offer some type of molecular advantage to the patient by recapitulating the high-volume high resource environment associated with superior outcomes? Although it might seem tempting to launch yet another analysis of cytokines, chemokines, stress hormones, microbiota, etc., here we posit that separating the parts from the whole of robotic surgery is unlikely to uncover such causative factors which are likely due to a combination of accumulated knowledge, technology, environmental control, and education that extend beyond the confines of the instrument itself (Fig. 1). A molecular analysis of this sort would likely suffer from major selection and attribution bias as robotic surgery and the surgeons that perform it tend to be those that have highly focused practices in which a limited repertoire of procedures is performed in this manner. That said, as the enabling technology of robotic surgery continues to improve in its instrumentation and intelligence capabilities and as training and on-site education advance surgeons to achieve a greater and faster road to mastery, one can imagine that there is more to this practice than simply the delivery of facile tissue dissection and reconstruction.

## Conclusion

While the advantage of robotic surgery may lie in its ability to inexorably embed intelligence and advanced instrumentation into its platform, much more may be at play here. Considering robotic surgery as simply “another tool” may belie its multiple advantages to make surgery ever more safe and effective via processes of teamwork, coordination, rehearsals, and training. Finally, the expectation that a peak performance is demanded by the very act of performing robotic surgery and expected by all members of the team may be one mechanism of its advantage that has been overlooked.
